# FOXO/TXNIP pathway is involved in the suppression of hepatocellular carcinoma growth by glutamate antagonist MK-801

**DOI:** 10.1186/1471-2407-13-468

**Published:** 2013-10-10

**Authors:** Fuminori Yamaguchi, Yuko Hirata, Hossain Akram, Kazuyo Kamitori, Youyi Dong, Li Sui, Masaaki Tokuda

**Affiliations:** 1 Departments of Cell Physiology, Faculty of Medicine, Kagawa University, 1750-1 Miki-cho, Kita-gun, Kagawa 761-0793, Japan

**Keywords:** MK-801, NBQX, FOXO, TXNIP, p27, G1 cell cycle arrest, HepG2, HuH-7, HLF

## Abstract

**Background:**

Accumulating evidence has suggested the importance of glutamate signaling in cancer growth, yet the signaling pathway has not been fully elucidated. N-methyl-D-aspartic acid (NMDA) receptor activates intracellular signaling pathways such as the extracellular-signal-regulated kinase (ERK) and forkhead box, class O (FOXO). Suppression of lung carcinoma growth by NMDA receptor antagonists via the ERK pathway has been reported. However, series of evidences suggested the importance of FOXO pathways for the regulation of normal and cancer cell growth. In the liver, FOXO1 play important roles for the cell proliferation such as hepatic stellate cells as well as liver metabolism. Our aim was to investigate the involvement of the FOXO pathway and the target genes in the growth inhibitory effects of NMDA receptor antagonist MK-801 in human hepatocellular carcinoma.

**Methods:**

Expression of NMDAR1 in cancer cell lines from different tissues was examined by Western blot. NMDA receptor subunits in HepG2, HuH-7, and HLF were examined by reverse transcriptase polymerase chain reaction (RT-PCR), and growth inhibition by MK-801 and NBQX was determined using the 3-(4,5-dimethylthiazol-2-yl)-2,5-diphenyltetrazolium bromide (MTT) assay. The effects of MK-801 on the cell cycle were examined by flow cytometry and Western blot analysis. Expression of thioredoxin-interacting protein (TXNIP) and p27 was determined by real-time PCR and Western blotting. Activation of the FOXO pathway and TXNIP induction were examined by Western blotting, fluorescence microscopy, Chromatin immunoprecipitation (ChIP) assay, and reporter gene assay. The effects of TXNIP on growth inhibition were examined using the gene silencing technique.

**Results:**

NMDA receptor subunits were expressed in all cell lines examined, and MK-801, but not NBQX, inhibited cell growth of hepatocellular carcinomas. Cell cycle analysis showed that MK-801 induced G1 cell cycle arrest by down-regulating cyclin D1 and up-regulating p27. MK-801 dephosphorylated Thr24 in FOXO1 and induced its nuclear translocation, thus increasing transcription of TXNIP, a tumor suppressor gene. Knock-down of TXNIP ameliorated the growth inhibitory effects of MK-801.

**Conclusions:**

Our results indicate that functional NMDA receptors are expressed in hepatocellular carcinomas and that the FOXO pathway is involved in the growth inhibitory effects of MK-801. This mechanism could be common in hepatocellular carcinomas examined, but other mechanisms such as ERK pathway could exist in other cancer cells as reported in lung carcinoma cells. Altered expression levels of FOXO target genes including cyclin D1 and p27 may contribute to the inhibition of G1/S cell cycle transition. Induction of the tumor suppressor gene TXNIP plays an important role in the growth inhibition by MK-801. Our report provides new evidence that FOXO-TXNIP pathway play a role in the inhibition of the hepatocellular carcinoma growth by MK-801.

## Background

Glutamate signaling is important for excitatory synaptic transmission in the central nervous system (CNS) and play a critical role in synaptic plasticity, a cellular mechanism for learning and memory [1,2]. In addition, glutamate receptors are expressed in non-neuronal cells throughout the body, including bone, skin, lung, liver, heart and kidney, and play distinct physiological roles in these tissues [3,4]. Interestingly, glutamate signaling is also involved in diseases such as cancer and neurological disorders [5-7]. In cancer cells, glutamate signaling pathways are dysregulated and glutamate is released from cancer cells, stimulating cell growth [8,9]. In pancreatic cancer, glutamate stimulates cell invasion and migration [10].

Glutamate receptors are divided into two major groups: ionotropic and metabotropic receptors [11,12]. The former group is further classified into three members based on their agonists: N-methyl-D-aspartic acid (NMDA), α-amino-3-hydroxyl-5-methyl-4-isoxazole-propionate (AMPA) and kainate receptors. Of these, the NMDA receptor forms a heterotetramer between the NR1 and NR2 subunits, including NR2A, NR2B, NR2C and NR2D, or NR3 subunits, such as NR3A and NR3B [13]. The NR1 subunit is necessary for calcium conductivity through the channel and the NR2 and NR3 subunits determine electrophysiological and pharmacological properties of the receptors. The different expression and distribution patterns of NR2 and NR3 subunits are responsible for the distinct functional properties of these receptors. In the brain, NMDA receptors are involved in the promotion of neuronal cell death or survival in addition to signal transduction via Ca^2+^ entry through NMDA receptors [14]. Synaptic NMDA receptor activity suppresses the induction of cell death-related genes such as Puma, Apaf-1 and FOXO [14]. Suppression of FOXO down-regulates target genes, including Bim, FasL and TXNIP, a tumor suppressor gene [15,16], whereas overexpression of FOXO1 enhances TXNIP promoter activity [17]. TXNIP was originally cloned as a vitamin D3 up-regulated protein (VDUP1) after 1, 25-dihydroxyvitamin D3 treatment of HL-60 cells [18]. Its expression is down-regulated in many tumor cells [19,20] and overexpression of TXNIP inhibits cancer cell growth [21].

NMDA receptors are expressed in many cancer cell lines and tumors, including glioma [22], oral squamous cell carcinoma [23], gastric cancer [24], prostate cancer [25] and osteosarcoma [26]. The expression pattern of the receptors differs between these cells [7]. NMDA receptors may play an important role in the growth of cancer cells, as there is some evidence that administration of glutamate antagonists inhibits cancer cell growth derived from brain, thyroid, colon, breast and lung tumors [27,28]. Although knowledge of the detailed mechanisms is limited, growth suppression of lung adenocarcinoma by the NMDA antagonist MK-801 via inhibition of the ERK pathway and induction of the tumor suppressor protein p21 and p53 has been reported [28].

As NMDA receptor signaling regulates cell death pathways such as FOXO in the CNS, we assumed that this pathway might be involved in the NMDA receptor signaling in cancer cells. In fact, FOXO pathway is involved in the regulation of cancer cell growth and FOXO is known as a tumor suppressor [29,30]. In the liver, FOXO pathway is important for cell proliferation [31] and the metabolism [32]. Therefore, we focused on the NMDA signaling and FOXO pathway in hepatocellular carcinomas. In this study, we confirmed the expression of NMDA receptors in HepG2, HuH-7, and HLF human hepatocellular cell lines. MK-801 suppressed the growth of these cells via G1 cell cycle arrest. Activation of the FOXO pathway and induction of TXNIP are involved in growth suppression by MK-801.This mechanism via the FOXO pathway is different from the previous report describing the importance of ERK pathway in the lung cancer treated with MK-801 [28].

## Methods

### Materials

MK-801, NBQX (2,3-Dioxo-6-nitro-1,2,3,4- tetrahydrobenzo[f]quinoxaline-7-sulfonamide), RNase A, DMEM and MEM alpha were purchased from Sigma (St. Louis, MO). 3-(4,5-dimethyl-2-thiazolyl)-2,5-diphenyl-2H-tetrazolum bromide (MTT) and propidium iodide were purchased from Wako (Tokyo, Japan). Fetal bovine serum (FBS) was purchased from MBL (Tokyo, Japan) and 1% penicillin-streptomycin was obtained from GE Healthcare (Chalfont, St. Giles, England).

### Cell culture

Human hepatocellular carcinoma cell lines, HepG2 and HuH-7 were purchased from Riken Cell Bank (Tsukuba, Japan) and HLF cell line was purchased from JCRB Cell Bank (Osaka, Japan). Human colon colorectal carcinoma cell line, HCT-116 was purchased from DS Pharma Biomedical Co. Ltd (Osaka, Japan). Human neuroblastoma cell line, SH-SY5Y was obtained from ATCC (Manassas, VA). These cells were maintained in DMEM supplemented with 10% (v/v) FBS and 1% penicillin-streptomycin at 37°C under a humidified atmosphere of 5% CO_2_.

### Cell viability assay

Cells were seeded into 96-well plates with 3 to 5 × 10^3^ cells/well in 0.1 ml of medium and cultured for 24 h. Various concentrations of MK-801 and NBQX were added to the culture medium and cells were further cultured. MTT solution (5 mg/ml in PBS) was added to each well and plates were incubated for an additional 4 h at 37°C. To solubilize the formazan crystal formed in viable cells, dimethylformamide-20% sodium dodecyl sulfate (pH 4.7) was added to each well, followed by incubation on a shaker at 37°C. Absorbance was measured on a microplate reader at 595 nm.

### RT-PCR and quantitative real-time PCR

In accordance with the manufacturer’s protocol, total RNA was purified from cultured cells using an RNAeasy mini prep kit (Qiagen, Hilden, Germany) and cDNA was synthesized using an Omniscript reverse transcriptase kit (Qiagen) with random hexamers. For RT-PCR analysis, sequences of the human NMDA receptor subunits were obtained from GENBANK and PCR primers were designed (Table 1). PCR was performed with these primers at 95°C for 3 min, followed by 40 cycles at 95°C for 10 s, 65°C for 30 s, and 72°C for 30 s with PCR Thermal Cycler Dice (Takara, Otsu, Japan). Amplified products were separated on 1.5% agarose gel, and images were obtained. PCR products were also subcloned into plasmid vectors using the Target clone kit (Toyobo, Tokyo, Japan) and sequences were confirmed using an ABI 3700 sequencer (Applied Biosystems, Foster City, CA). Real-time quantitative PCR was carried out using Taqman gene expression assay primers and a 7300 real-time PCR system (Applied Biosystems). The assay ID of Taqman probe is Hs00197750_m1 for TXNIP and Hs99999903_01 for β-actin. Each reaction was performed in duplicate. The β-actin gene was used for normalization across assays and runs, and the threshold value (Ct) for each sample was used to determine gene expression levels.

**Table 1:** Primer pairs used for RT-PCR experiment

**Gene name**		**Sequence (5′ → 3′)**	**Genbank accession**
NR1:	sense	TATGGAGAAGCACAACTACGAGAG	NM_000832
	antisense	CGAGCAGCAGGACCCATCAGTGT	
NR2A	sense	CGGGTATGATTTCTTCTGGATTGTCCC	NM_000833
	antisense	GGGTGACGATGCTGAGATGGTTGT	
NR2B	sense	AGCCCCATCATTCTTCTTTACTGTACCAAG	NM_000834
	antisense	CCTTTCCCACTTCCTCTCCTTGTTCAG	
NR2C	sense	CTTTGTGGAGACGGGCATCAGTGT	NM_000835
	antisense	GGCGAGGAAGATGACAGCAAAGAA	
NR2D	sense	CCTGCTGCGTGATTATGGTTTCCT	NM_000836
	antisense	GAGGGCTGTGGGTTCGGTTGA	
NR3A	sense	CTTTTTAGCAGCCTCCATAGCAGTAATGA	NM_133445
	antisense	TCATACAGAGTGAGGAAGACGGCAGTG	
NR3B	sense	GTATCAACTCCGCCCGCTCACAG	NM_138690
	antisense	AGAGGATGGCGTAGCACAGGTTGA	
β-actin	sense	CTAACTTGCGCAGAAAACAAGAT	NM_001101
	antisense	TTCCTGTAACAACGCATCTCATA	

Sequences of the human NMDA receptor subunits were obtained from GENBANK, and PCR primers were designed.

### Cell cycle analysis

Cells were cultured in 10-cm dishes with or without 250 μM of MK-801, and were harvested after 72 h by trypsinization (0.25% trypsin / 1 mM EDTA), washed twice with ice-cold PBS and fixed in 1 ml of 70% ethanol (1 × 10^6^ cells/sample) for 2 h at 4°C. Cells were washed twice with ice-cold PBS and incubated in 1 ml PBS containing 50 μg propidium iodide and 200 μg RNase A for 30 min at 37°C in the dark. Flow cytometric analysis was performed with FACSEpics XL flow cytometer (Beckman Coulter, Fullerton, CA). The effects of MK-801 on cell cycle were determined by changes in the percentage cell distribution at each phase of the cell cycle, and were analyzed using System II software (Beckman Coulter).

### Western blot analysis

Cells were washed with PBS and scraped into lysis buffer (50 mM Tris–HCl pH 7.5, 0.5% Triton-X100, 0.5% Tween20) containing protease inhibitors (Sigma), and were sonicated. Samples were centrifuged for 10 min at 15,000 rpm at 4°C and the supernatants were collected. Proteins were separated on SDS-PAGE gels, transferred to nitrocellulose membranes and blocked with 5% (w/v) non-fat dried milk in TTBS, followed by incubation with anti-cyclin D1 (MBL), anti-cyclin E (MBL), anti-cyclin-dependent kinase (CDK) 2 (Santa Cruz Biotechnology, Santa Cruz, CA), anti-CDK4 (MBL), anti-p53 (Santa Cruz Biotechnology), anti-p27 (Cell Signaling Technologies, Beverly, MA), anti-p21 (Cell Signaling Technologies), anti-TXNIP (Santa Cruz Biotechnology), anti-phospho-FOXO1/FOXO3a (Thr24/Thr32), anti-NMDAR1 (Millipore, Billerica, MA) and anti-FOXO1 (Cell Signaling Technologies) in Can Get Signal Immunoreaction Enhancer Solution (TOYOBO, Tokyo Japan), or with anti-β-actin antibody (Sigma) in 5% (w/v) non-fat dried milk in TTBS. Membranes were washed and probed with horseradish peroxidase (HRP)-conjugated anti-rabbit or anti-mouse IgG (GE Healthcare), and signals were detected using Immobilon Western chemiluminescent HRP substrate (Millipore).

### Fluorescence microscopy

The coding region of FOXO1 was cloned into pAcGFP-N1 vector (Clontech). HepG2 cells were seeded into 35 mm dish and FOXO1-pAcGFP-N1 plasmid was transfected using FuGENE HD transfection reagent (Roche, Indianapolis, IN) in accordance with the manufacturer’s protocol. After 24 h, medium was replaced with PBS and the temperature was kept at 37°C in a heat chamber. GFP-tagged FOXO1 protein was visualized with Olympus LX71 microscope (Olympus, Tokyo, Japan) in the presence or absence of MK-801.

### Reporter gene assay

The promoter region of human TXNIP containing a conserved FOXO binding site (between -478 and -260 nucleotides from the start of protein coding region) was obtained by PCR from human genomic DNA and subcloned into the PGL4.6 reporter plasmid (Promega, Madison, WI). Mutation construct with a destroyed FOXO consensus sequence (FOXO-Mut) was created by PCR using the PrimeSTAR Mutagenesis Prime Kit (Takara). HepG2 (3 × 10^4^ cells/well) cells were seeded into 24-well plates and reporter plasmid (500 ng/well) was co-transfected with pGL4.74 control reporter plasmid (50 ng/well) using FuGENE HD transfection reagent in accordance with the manufacturer’s protocol. After 24 h of MK-801 treatment, cells were lysed and luciferase activity was measured using the Dual-Glo Luciferase Assay System (Promega) with Luminoskan (Labosystems, Tokyo Japan). Variations in transfection efficiency between samples were normalized against the luminescence of a control reporter.

### ChIP (chromatin immunoprecipitation) assay

The ChIP assay was performed using a ChIP-IT High Sensitivity Kit (Active Motif, Carlsbad, CA) according to the manufacturer’s instructions. Briefly, 6 × 10^6^ cells were fixed and shared. ChIP reactions were performed on 5 μg of prepared chromatin using 5 μl of anti-phospho-FOXO antibody. The immune complexes were then collected with the addition of Protein G agarose beads, followed by several washes with appropriate buffers, according to the manufacturer’s instructions. Chromatin-associated proteins were digested with proteinase K (10 mg/ml), and the immunoprecipitated DNA was recovered by spin columns. PCR was performed using input or immunoprecipitated DNA. The primers used for ChIP were as follows: 5′-CACGCGCCACAGCGATCTCACTGA-3′ (-472 to -449, sense) and 5′-AGATCCGATCTCCACAAGCACTCC-3′ (-284 to -261, antisense). The PCR product was separated in 1.5% agarose gel.

### Gene knock-down by small interfering RNA (siRNA)

HepG2 cells (5 × 10^4^ cells/well) were cultured in a 96-well plate. For the knock-down experiment, 5 nM TXNIP siRNA (siRNA1: 5′-UGCUCGAAUUGACAGAAAATT-3′, siRNA2: 5′-GUGGAGGUGUGUGAAGUUATT-3′, Cosmo Bio Co. Ltd., Tokyo, Japan) or negative control siRNA (Qiagen) was transfected using the Hiperfect transfection reagent (Qiagen) in accordance with the manufacturer’s protocol. After 24 h, MK-801 (125 or 250 μM) was added to the medium and cells were further incubated for 48 h. Cell viability was examined using the MTT method.

### Statistical analysis

Data are presented as means ± SD of at least three independent experiments. Differences between groups were analyzed by one-way analysis of variance with Bonferroni post-hoc analysis or unpaired t-test.

## Results

### Expression of NMDA receptor subunits in hepatocellular carcinoma cell lines

**Figure 1:**
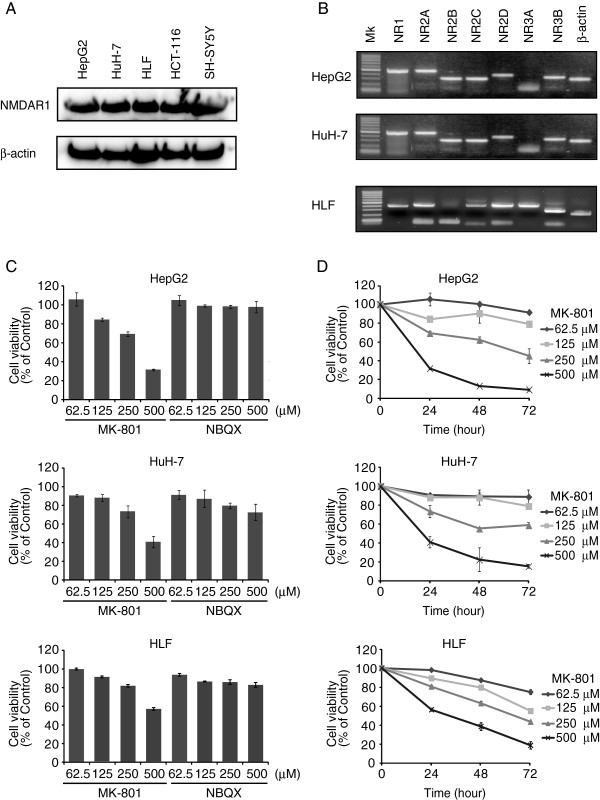
**MK801 inhibited growth of hepatocellular carcinoma cells. A**: Cell lysates prepared from hepatocellular carcinoma (HepG2, HuH-7, and HLF), colon colorectal carcinoma (HCT-116), and neuroblastoma (SH-SY5Y) were separated on 12.5% SDS-PAGE and western blot analysis was performed with anti-NMDAR1 or anti-β-actin antibody. **B**: Total RNA was purified from cultured cells and cDNA was synthesized with random hexamers. PCR was performed with NMDAR subtype or β-actin specific primers Amplified products were separated on 1.5% agarose gels. **C**: Cells were seeded into 96-well plates and different concentrations of MK-801 or NBQX were added to culture medium for 24 h. Cell viability was measured by MTT assay. Each bar represents the mean ± SD of three replicates. **D**: Various concentrations of MK-801 were added to the medium and cells were cultured for 72 h. Cell viability was measured by MTT assay. Each point represents the mean ± SD of three replicates.

First, we compared the expression of NMDAR1 receptor by western blot analysis using cells lines from different origins including SH-SY5Y (neuroblastoma), HCT-116 (colon colorectal carcinoma), and three hepatocarcinomas (HepG2, HuH-7, and HLF). We detected a substantial amount of NMDAR1 expression in all cell lines examined (Figure 1A). This result is similar to the previous report describing the NMDAR1 expression in peripheral tissues including heart, kidney, liver, and spleen [33]. Next, we analyzed the expression of each receptor subunit in HuH-7, HepG2, and HLF cell lines. Specific primers for NMDA receptor subunits were designed (Table 1) and RT-PCR was carried out. In HepG2 and HuH-7 cell lines, expression of NR1, NR2 (R2A, R2B, R2C and R2D) and NR3B subunits was confirmed, whereas NR3A expression was not detected (Figure 1B). We could not detect NR2B subunit in HLF cells, but NR3A subunit was expressed.

### NMDA receptor antagonist MK-801 inhibits HepG2, HuH-7, and HLF cell proliferation

As these hepatocellular carcinoma cell lines expressed NMDA receptor subunits, the effects of the NMDA antagonist, MK-801 on proliferation was examined and compared to that of the non-NMDA receptor antagonist, NBQX. Increasing concentrations of these antagonists were applied, and cell viability was examined by MTT assay after 24 h (Figure 1C). MK-801 inhibited the growth of these cells in a dose-dependent manner. Treatment with MK-801 (500 μM) decreased cell viability to 31.6% ± 0.9% (HepG2), 40.9% ± 5.8% (HuH-7), and 56.6% ± 1.5% (HLF) (n = 3 each). These results indicated that the NMDA receptors in these cell lines were functional and may regulate growth. In contrast, NBQX showed no clear effects on the proliferation of either cell lines, although a weak inhibitory effect was observed in HuH-7 cells. Cell viability was examined up to 72 h after drug treatment (Figure 1D). After 72 h, growth suppression with 500 μM MK-801 reached 9.0% ± 1.4% with HepG2, 15.5% ± 2.2% with HuH-7 cells, and 19.3% ± 3.8% with HLF cells (n = 3 each).

### MK-801 inhibits G1/S transition of the cell cycle

**Figure 2:**
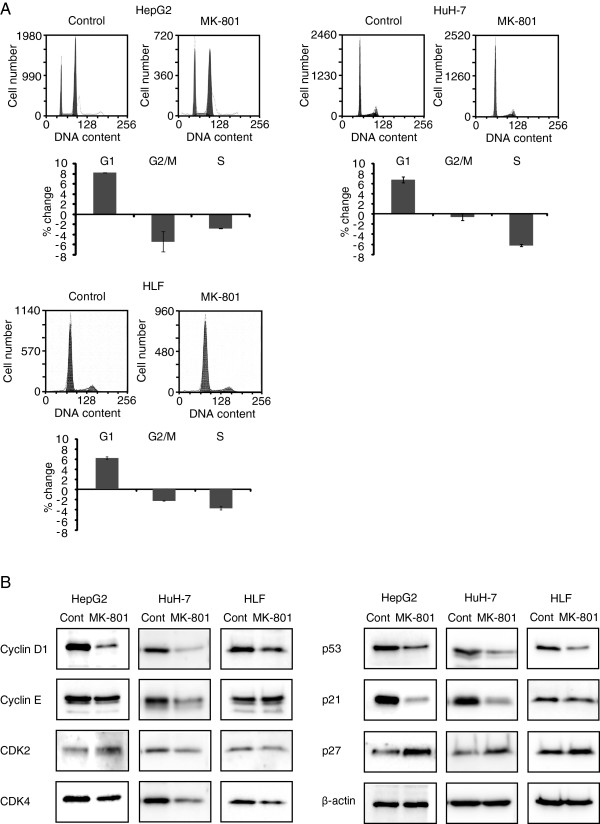
**Cell cycle analyses by flow cytometry and Western blotting. A**: Hepatocellular carcinoma cells were cultured in 10-cm dishes with or without 250 μM MK-801 and were harvested after 72 h by trypsinization and fixed in 70% ethanol. Cells were incubated in 1ml PBS containing 50 μg propidium iodide and 200 μg RNase A. Flow cytometric analysis was performed with a FACSEpics XL flow cytometer. Each bar represents the mean ± SD of three replicates. **B**: Cell lysates from control or MK-801 (250 μM) treated cells were prepared and proteins were separated on SDS-PAGE gels and transferred onto nitrocellulose membranes. Western blot analysis was performed with indicated antibodies.

In order to clarify the mechanism of MK-801-induced inhibition of cell growth, we analyzed cell cycle progression in both cell lines by flow cytometry (Figure 2A). When compared with controls, MK-801 increased the percentage of HepG2 cells in G1 phase (8.2% ± 0.1%) and decreased that in G2/M and S phases (-5.4% ± 2.0% and -2.8% ± 0.1%, respectively). In HuH-7 cells, MK-801 also increased the percentage in G1 phase (6.8% ± 0.6%) and decreased that in G2/M and S phases (-0.6% ± 0.7% and -6.3% ± 0.2%, respectively). In HLF cells, MK-801 increased the percentage of cells in G1 phase (6.1% ± 0.2%) and decreased that in G2/M and S phases (-5.4% ± 2.0% and -2.3% ± 0.1%, respectively). These results indicate that MK-801 inhibits G1/S cell cycle transition. The expression level of cell cycle-regulating proteins was then examined by Western blot analysis (Figure 2B). In these cells, down-regulation of cyclin D1, p53 and p21 was observed. In addition, expression of cyclin E, CDK2 and CDK4 was down-regulated in HuH-7 cells. Interestingly, expression of p27 was up-regulated in all cell lines.

### MK-801 induces TXNIP expression

**Figure 3:**
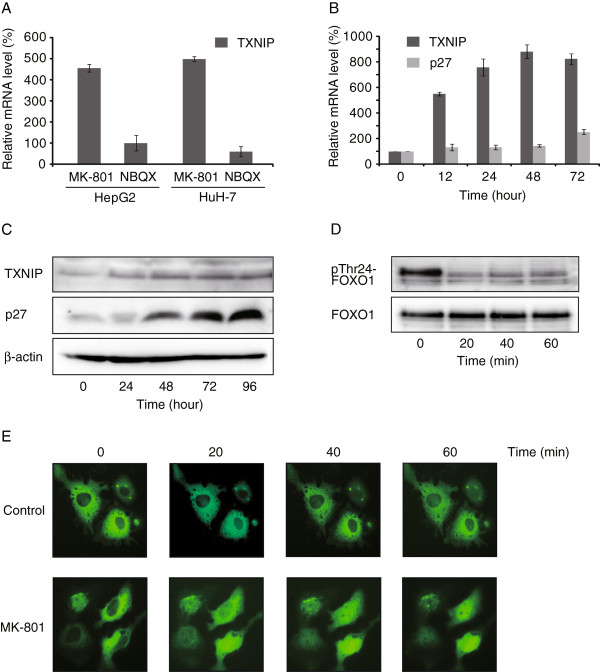
**Activation of FOXO pathway and TXNIP induction. A**: HepG2 and HuH-7 cells were treated with 250 μM MK-801 or NBQX for 12 h. cDNA was synthesized and real-time quantitative PCR was carried out using Taqman gene expression assay primers. Each reaction was performed in duplicate. The β-actin gene was used to normalize across assays and runs, and the threshold value (Ct) for each sample was used to determine gene expression levels. Each bar represents the mean ± SD of at least three replicates. **B**: HepG2 cells were treated with 250 μM MK-801 and expression of TXNIP and p27 was measured by real-time quantitative PCR for 72 h. Each bar represents the mean ± SD of at least three replicates. **C**: HepG2 cells were treated with 250 μM MK-801 for 0 to 96 h and Western blot analysis was performed using indicated antibodies. **D**: HepG2 cells were treated with 250 μM MK-801 for 0 to 60 min and Western blot analysis was carried out using indicated antibodies. The molecular weight of dephosphorylated band corresponded to Thr24 of FOXO1 **E**: FOXO1-pAcGFP-N1 plasmid was transfected to HepG2 cells and treated with or without 250 μM of MK-801. Nuclear translocation of FOXO1-GFP protein was observed with Olympus LX71 microscope.

As NMDA receptors regulate cell death via several pathways, including FOXO [14], we examined the effects of MK-801 and NBQX on expression of the FOXO target gene TXNIP by real-time PCR (Figure 3A). After 12 h, MK-801 (250 μM) induced significant TXNIP expression (453.7% ± 17.5% with HepG2 and 496.2% ± 23.8% with HuH-7 cells). In contrast, NBQX showed no clear effect on TXNIP expression. The induction of TXNIP mRNA reached 900% after 48 h of MK-801 (250 μM) treatment (Figure 3B). TXNIP is known to induce cell cycle arrest by stabilizing p27 protein via inhibition of JAB1 [34]. Therefore, we examined the time-course changes of p27 mRNA and protein levels. After 48 h, protein levels of p27 were clearly elevated by MK-801 treatment (Figure 3C). In contrast, induction of p27 mRNA was slower and reached 251.9% ± 21.4% after 72 h (Figure 3B). Transcriptional activity of FOXO1 is regulated by the phosphorylation state of Thr24, Ser256 and Ser319 [35]. Western blot analysis showed that MK-801 treatment of HepG2 cells rapidly (within 20 min) dephosphorylated phospho-FOXO1. The molecular weight of the dephosphorylated band corresponded to Thr24 of FOXO1 (Figure 3D). The dephosphorylation of FOXO1 induces the nuclear translocation and increases the expression of FOXO1 target genes [35]. MK-801 treatment of HepG2 cells enhanced the nuclear localization of FOXO1 (Figure 3E).

### Regulation of TXNIP expression by FOXO and role of TXNIP in growth inhibition

**Figure 4:**
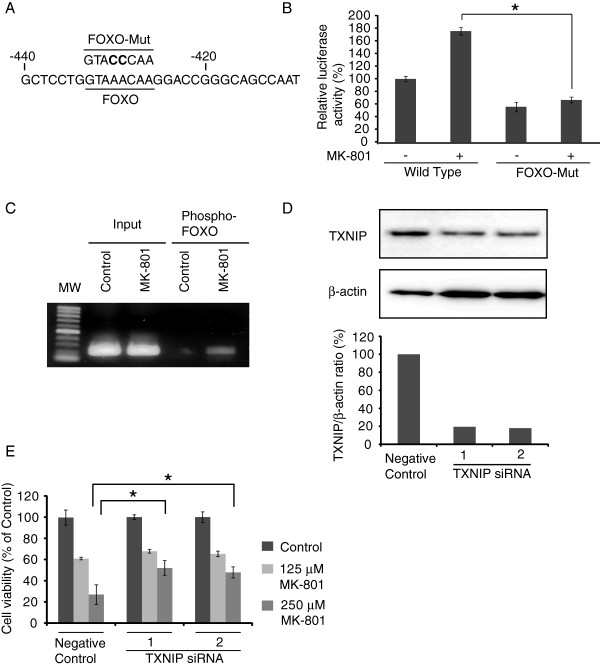
**Regulation of TXNIP expression by FOXO and role of TXNIP in growth inhibition. A**: The promoter region of human TXNIP containing the FOXO binding site (between -478 and -260 nucleotides from the start of the protein coding region) is shown. FOXO-Mut: The consensus sequence of FOXO binding site was destroyed by two nucleotide mutations. **B**: Reporter gene plasmid containing the wild-type or FOXO-Mut promoter fragment and pGL4.74 control plasmid was co-transfected into HepG2 cells. After 24 h of MK-801 (250 μM) treatment, luciferase activity was measured. Each bar represents mean ± SD of three replicates. *P < 0.05. **C**: HepG2 cells were treated with or without MK-801 (250 μM). ChIP reaction was performed on 5ug of prepared chromatin using anti-phospho-FOXO antibody. PCR was carried out with input or immunoprecipitaed DNA using specific primers flanking the FOXO binding site in the TXNIP promoter region. **D**: The gene knock-down efficiency of two TXNIP siRNAs as well as negative control on HepG2 cells was examined by Western blot. TXNIP/β-actin ratio was calculated from the density of each band. **E**: HepG2 cells were cultured on a 96-well plate and 5 nM of TXNIP or negative control siRNA was transfected. After 48 h of MK-801 (125 or 250 μM) treatment, cell viability was examined by the MTT assay. Data were statistically analyzed by one-way analysis of variance and Bonferroni post-test. *P < 0.05.

The promoter region of TXNIP contains a conserved consensus sequence, ′GTAAACAA′, of the FOXO binding site that regulates TXNIP transcription [17]. To analyze the effects of MK-801 on TXNIP expression, we constructed a luciferase reporter gene plasmid by subcloning the promoter region of human TXNIP (wild type) or FOXO-Mut by destroying the FOXO binding site (Figure 4A). These plasmids were transfected into HepG2, and the effects of MK-801 on TXNIP promoter activity were examined (Figure 4B). MK-801 increased the promoter activity when the wild-type promoter region was transfected, but failed to activate the FOXO-Mut promoter.

To confirm the binding of FOXO to TXNIP promoter, we performed ChIP assay on HepG2 cells treated with or without MK-801 (Figure 4C). When cells were treated with MK-801, primers flanking a FOXO consensus binding site specifically amplified DNA sequences immunoprecipitated by anti-phospho-FOXO antibody, indicating that FOXO1 is able to bind the TXNIP promoter in vivo, which supports the result of the increase of luciferase activity in Figure 4B. Next, we examined the effects of TXNIP on MK-801-induced cancer cell growth inhibition. We tested two different TXNIP siRNAs to eliminate the possibility of off-target effects. The western blot showed that both TXNIP siRNAs effectively knocked down the TXNIP expression (Figure 4D). These TXNIP or negative control siRNA were transfected into HepG2 cells and the effects of MK-801 were analyzed by the MTT method. TXNIP siRNAs significantly attenuated the effects of cell growth inhibition by MK-801 (P < 0.05) (Figure 4E).

## Discussion

Our study demonstrated that the NMDA receptor antagonist MK-801 inhibited growth of HepG2, HuH-7, and HLF human hepatocellular carcinomas, and that the effects were induced by the functional expression of NMDA receptors in these cancer cells. The expression pattern of NMDA receptor subtypes was similar between HepG2 and HuH-7 cell lines. HLF cells lack NR2B, but express NR3A subtypes. MK-801 inhibited the growth of these cells in a dose dependent manner. In contrast, NBQX, an AMPA receptor antagonist, showed no effect on HepG2 cell growth, whereas weak inhibition was observed in HuH-7 cells. Although the involvement of AMPA receptors in cancer cell growth in glioblastoma, colon and lung carcinoma has been reported [28,36], the expression of AMPA receptors in liver or hepatocellular carcinoma is unknown. Further study of AMPA receptor signaling in hepatocellular carcinoma could be useful in interpreting the results of NBQX.

The antiproliferative effects of MK-801 arise from cell cycle arrest caused by modulation of cell cycle-regulating gene expression. A previous study using lung carcinoma A549 cells demonstrated that MK-801 suppresses cancer cell growth by down-regulating cyclin D1 and up-regulating p53 and p21 expression [28]. Cyclin D1 is essential for cell cycle progression in G1 [37] and p21 is important for p53-mediated G1 arrest in human cancer cells [38]. Silencing p21 ameliorates the antiproliferative effects of MK-801, thus suggesting that p21 is a key regulator of cell cycle arrest by MK-801. The expression levels of these cell cycle-regulating genes could be modulated by inhibiting the ERK pathway [28]. Interestingly, in the HepG2, HuH-7, and HLF cell lines, p53 and p21 were down-regulated and p27 was up-regulated by MK-801. The level of p27 controls G1 cell cycle progression and increased levels of p27 induce cell cycle arrest at the G1 phase [39]. Up-regulation of p27 by MK-801 may contribute to G1 cell cycle arrest in these cells.

NMDA receptor signaling is linked to the FOXO pathway as well as to the ERK pathway [14]. FOXO1 is a member of forkhead family of transcription factors and regulates the expression of a number of genes that play critical roles in cell cycle and apoptosis [40]. In addition, FOXO plays a tumor suppressor role in various cancers [29,30]. FOXO induces G1 cell cycle arrest by inducing p27 [41,42] and suppressing cyclin D1 expression [43]. TXNIP is identified as another FOXO target gene [44] and MK-801 increases TXNIP promoter activity [28]. Transcriptional activity of FOXO1 is regulated by the phosphorylation state of Thr24, Ser256 and Ser319 and phosphorylation at these sites suppress transactivation and promotes the redistribution of FOXO1 outside the nucleus [35]. Activation of FOXO1 by Thr24 dephosphorylation may induce down-regulation of cyclin D1 and up-regulation of p27. In addition, transcriptional activation of the FOXO target gene TXNIP acts as a tumor suppressor. TXNIP stabilizes p27 protein by inhibiting its degradation via JAB1 [34] and induces G1 cell cycle arrest [21]. This may explain the differences in transcript and protein levels of p27 in the time course study. Our study indicates that the TXNIP-FOXO axis contributes to cell cycle arrest and is important for MK-801-mediated hepatocellular carcinoma growth inhibition. This mechanism could be common in hepatocellular carcinomas examined, but other mechanisms such as ERK pathway could exist in other cancer cells as reported in lung carcinoma cells [28].

## Conclusions

In conclusion, this study provides insight into the role of the FOXO signaling pathway on growth inhibition by the NMDA receptor antagonist MK-801 in hepatocellular carcinoma. In addition, our data indicate that FOXO activates transcription of the tumor suppressor gene TXNIP, which contributes to the growth inhibitory effects of MK-801. Our report provides new evidence that FOXO-TXNIP pathway play a role in the inhibition of the hepatocellular carcinoma growth by MK-801. Future research should therefore consider members of this pathway as potential targets for the treatment of hepatocellular carcinoma through NMDA receptor signaling.

## Abbreviations

AMPA: α-amino-3-hydroxyl-5-methyl-4-isoxazole- propionate; CDK: Cyclin-dependent kinase; ChIP: Chromatin immunoprecipitation; CNS: Central nervous system; ERK: Extracellular-signal-regulated kinase; FOXO: Forkhead box, class O; NBQX: 2,3-Dioxo-6-nitro-1,2,3,4- tetrahydrobenzo[f]quinoxaline-7-sulfonamide); NMDA: N-methyl-D-aspartic acid; MTT: (3-(4, 5-dimethylthiazol-2-yl)-2, 5-diphenyltetrazolium bromide); RT-PCR: Reverse transcription-polymerase chain reaction; TXNIP: Thioredoxin interacting protein.

## Competing interests

The authors declare that they have no competing interests.

## Authors’ contributions

FY, YH, HM, KK, YD and LS performed the experiments. MT participated in the design of the study and helped draft the manuscript. All authors read and approved the final manuscript.

## Pre-publication history

The pre-publication history for this paper can be accessed here:

http://www.biomedcentral.com/1471-2407/13/468/prepub
