# Editorial Expression of Concern: FOXO/TXNIP pathway is involved in the suppression of hepatocellular carcinoma growth by glutamate antagonist MK-801

**DOI:** 10.1186/s12885-022-09764-1

**Published:** 2022-06-17

**Authors:** Fuminori Yamaguchi, Yuko Hirata, Hossain Akram, Kazuyo Kamitori, Youyi Dong, Li Sui, Masaaki Tokuda

**Affiliations:** Departments of Cell Physiology, Faculty of Medicine, Kagawa University, 1750-1, Miki-cho, Kita-gun, Kagawa 761-0793 Japan

**BMC Cancer 13, 468 (2013)**

**https://doi.org/10.1186/1471-2407-13-468**

The Editor is issuing an editorial expression of concern to alert readers that concerns have been raised about possible figure duplication in Figure 1B. Panels HepG2 and HuH-7 appear to be nearly identical. The authors no longer have access to raw data and therefore cannot provide a replacement image. The Editor advises readers to interpret the results of this section of the article with caution [1].

Authors Fuminori Yamaguchi and Kazuyo Kamitori agree to this Editorial Expression of Concern. Authors Hossain Akram and Masaaki Tokuda have not responded to any correspondence from the editor or publisher about this Editorial Expression of Concern. The editor was not able to obtain a current email address for authors Yuko Hirata, Youyi Dong and Li Sui.

## References

[1] Yamaguchi F, Hirata Y, Akram H (2013). FOXO/TXNIP pathway is involved in the suppression of hepatocellular carcinoma growth by glutamate antagonist MK-801. BMC Cancer.

